# Supplementary material for: Genetic Ablation of MiR-22 Fosters Diet-Induced Obesity and NAFLD Development
Source: J Pers Med. 2020 Oct 14;10(4):170. doi: 10.3390/jpm10040170 (PMC7711493; doi:10.3390/jpm10040170)
Supplement: Supplementary file 1 [file jpm-10-00170-s001.pdf]

SUPPLEMENTARY MATERIALS AND METHODS

Genetic ablation of miR-22 fosters diet-induced obesity and NAFLD development.

Monika Gjorgjieva[1*], Cyril Sobolewski[1*], Anne-Sophie Ay[1*], Daniel Abegg[2], Marta Correia de Sousa[1], Dorothea Portius[1], Flavien Berthou[1], Margot Fournier[1], Christine Maeder[1], Pia Rantakari[3], Fu-Ping Zhang[3], Matti Poutanen[3], Didier Picard[4], Xavier Montet[5], Serge Nef[6], Alexander Adibekian[2], Michelangelo Foti[1,7]

[1]Department of Cell Physiology and Metabolism, Faculty of Medicine, University of Geneva, Switzerland.

[2]Department of Chemistry, The Scripps Research Institute, 130 Scripps Way, Jupiter, Florida 33458, United States.

[3]Institute of Biomedicine, Research Centre for Integrative Physiology and Pharmacology, and Turku Center for Disease Modeling, University of Turku, Finland.

[4]Department of Cell Biology, Faculty of Science, University of Geneva, Switzerland.

[5]Department of Radiology, Faculty of Medicine, University of Geneva, Switzerland.

[6]Department of Genetic Medicine and Development, Faculty of Medicine, University of Geneva, Switzerland.

[7]Diabetes Center, Faculty of Medicine, University of Geneva, Switzerland.

** MG, CS and ASA equally contributed to this work.*

miR-22KO mice generation

Targeting construct

BAC clone (CH29-40010) containing the mouse *Mir22* gene was obtained from BACPAC Resources Center (Children's Hospital Oakland Research Institute, Oakland (CHORI), California, USA). A 9066 bp fragment of the *Mir22* gene containing exon 1 was subcloned into the minimal vector, pACYC177 (New England Biolabs, MA, USA) by Red/ET recombination method according to the manufacturer's instructions (Gene Bridges GmbH, Germany). PCR product used in subcloning was amplified from the pACYC177-plasmid using the primers 5'- **GTCAATGAAGTTCTTACGGTGACACTCAGCTTCCCAGAATGCAGCGCGCT** *GGCAGACCTCAGCGCTAG*-3' and 5'-**AGTACCCAGTGGGAGAATGGATC TAGGTCTGGGAGAAATTGACCTTAGC***TGAAGACGAAAGGGCCTC*-3' where homology arms to the *Mir22* gene are indicated in bold, and sequences homologues for ampicillin resistance (amp) gene and ori of the pACYC177-plasmid are in italics. Using the Red/ET recombination method we then further inserted a floxed PGK-Neo cassette (Gene Bridges) into the modified pACYC177-plasmid, into the 5' flanking region of exon 1 of the *Mir22* gene. The fragment used was generated by PCR and contained 50 bp *Mir22* homology arms for proper insertion. Primers were 5'- **GAAGATGCTGGGCTCCTTAGCAGGTTGCCAAGAGTTGCCAGCTCCTAGTA GATCT***GAAGCAGGGATTCTGCAAACC*-3' and 5'- **GAATAAGAAGCAAGAGGCTTCTAAAGCACTTGCTTTGTGCCCCTTTACAGG***G CGGATTTGTCCTACTCAGG*-3' where homology arms to the target gene are indicated in bold and sequences homologues for pGK-loxP-NEO are shown in italics. In order to leave a single loxP site at the targeted site, Cre-mediated recombination was carried out *in vitro* in *E.coli.* (New England Biolabs, MA, USA) by transforming the plasmid into E.coli. expressing Cre-recombinase. After transformation, cells were plated onto selection plates including 30μg/μl ampicillin, and ampicillin-resistant colonies were picked. The appearance of Cre-recombination was assessed by restriction enzyme analyses of plasmid DNA and by PCR. A second loxP site was introduced into the 3' flanking region of exon 1 with PGK-FTR-Neo-FRT-loxP cassette (Gene Bridges) containing 50 bp *Mir22* homology arms amplified by PCR. The primers were 5'- **GTGACTCCACCTCCCCAAGATTAGTTGATGTCTGTGTGGCAGTG GGAAGA***AATTAACCCTCACTAAAGGGC* -3' and 5'- **GGCAGGCCTACAAGGAAGTCTCAGTAAACAGACAGACTTTTCCTTCTATT** *TAATACGACTCACTATAGGGCTCGAG* -3'. The validity of the final targeting construct was confirmed by restriction enzyme digestion and sequencing.

Gene targeting in ES cells and blastocyst injection

G4 embryonic stem cells (derived from mouse 129S6/C57BL/6Ncr) were cultured on neomycin-resistant primary embryonic fibroblast feeder layers, and 10^7 cells were electroporated with 30 µg of the linearized targeting construct. After electroporation, the cells were plated on 100-mm culture dishes and exposed to G418 (300 µg/ml; Sigma). Colonies were picked after 7-9 days selection and grown on 96-wells plate. DNA isolated from ES cell clones targeted with the construct was screened by long-range PCR, which produced a 2693 bp fragment with a primer pair corresponding to 5' end of the *Mir22* gene (M22ges1: TCCCTATACCCCGAAGACCT) and 3'end of PGK-Neo cassette (Neoneoan: GGCGGATTTGTCCTACTCAGG). Up to 288 clones were picked and screened by PCR, and two clones containing a correct size fragment were then confirmed by sequencing. In order to delete the Neo-cassette included in the targeting vector, the once targeted ES cells were re-electroporated with the plasmid expressing the Frt-recombinase (pCAGGS-Flpo-IRES-puro). After electroporation, the cells were plated on 100-mm culture dishes and colonies were picked up after 3-5 days growth and grown on 96-wells plate. Four correct clones were identified from the 96 clones analyzed, and the right clones were further confirmed by sequencing.

The targeted ES cells with the two loxP sites inserted were injected into C57BL/6N mouse blastocysts to generate chimeric mice. Germline transmission was achieved by crossbreeding male chimeras with C57BL/6N females. C57BL/6N mice used as blastocyst donors were obtained from Charles River Laboratories (Willmington, MA). The mice were maintained in a specific pathogen-free stage at Central Animal Laboratory at the University of Turku. All studies carried out with the mice were approved by The Finnish ethical committee for experimental animals, complying with international guidelines on the care and use of laboratory animals.

Generation of constitutive miR-22KO mice

To generate constitutive mutants for *Mir22*, we disrupted *Mir22* in the male germline by combining a conditional *Mir22 floxed* allele with a germ-cell specific *Ngn3* promoter-driven transgenic Cre line(1). The genotype of male gametes produced by this double transgenic line was miR-22 KO. Mice heterozygous for the *Mir22* constitutive KO were viable and fertile and subsequent matings with these animals resulted in the expected ratio of 25% homozygous mutant mice, hereafter referred to as "miR-22KO".

Mice with constitutive *Mir22* deletion and CTL mice were first generation from littermate couple in open cages in non-SPF facilities.

Quantitative PCR (RT-qPCR) analyses

Total RNA from the liver was extracted with Trizol reagent (Ambion, Thermo Scientific, USA) according to the manufacturer instructions. For microRNAs, reverse transcription was performed with 500ng of total RNA using Poly A polymerase and the Protoscript II Retrotranscriptase (New England Biolabs, USA), Sno-202, and miR-16 were used to normalize gene expression.

One microgram RNA was used for cDNA synthesis with high capacity RNA to cDNA kit (Applied Biosystems Life Technologies Europe, Switzerland) and PCR amplification was performed with SYBR Green (Roche, Switzerland) in a StepOneTM PCR system (Applied Biosystems, Life Technologies Europe, Switzerland). Cyclophilin A was used to normalize gene expression. Primer sequences are listed in Supplementary Table 1.

Biochemical analyses in plasma and tissue

Plasma levels of triglycerides (TG), cholesterol, and transaminases were determined by an automated Abott Architect analyzer (Abott Architect, Paris, France). Non-esterified fatty acids measurement was assessed using Cayman Free Fatty Acid Assay Kit. Insuline and C-peptide were determined using mouse insulin Elisa (Mercodia, Sweden) and mouse C-peptide Elisa (Merck-Millipore, USA) respectively.

Triglycerides (TG) extraction from cryo-preserved hepatic samples or HepG2 cells was performed by hexane/isopropanol. TG content was then measured by colorimetric enzymatic analysis with commercial kits (TG Roche/Hitachi, Roche, Switzerland).

Metabolic phenotyping, EchoMRI analyses, and CT-scan imaging

An EchoMRI-700 quantitative nuclear magnetic resonance analyser (Echo Medical Systems, USA) was used to measure total fat and lean mass. Distribution, volume, and weight of fat depots were analysed by a multidetector CT-scan (Discovery 750 HD, GE Healthcare, Milwaukee, USA) and quantified with Osirix software (Switzerland). Metabolic phenotyping of mice was performed using a LabMaster system (GmbH Berlin, Germany) after 5 days of adaptation prior to recording.

Glucose tolerance tests

Glucose tolerance tests (GTT) were performed as previously described (2). Briefly, mice were starved overnight and a glucose load of 1.5g/kg was administered i.p. Blood samples were collected at indicated times, and the glucose level measured using Glucotrend Active (Roche, Switzerland).

Histological Analyses

Liver samples were fixed in 4% paraformaldehyde and after dehydration and embedment in paraffin, 5-µm-thick sections were stained with hematoxylin/eosin (H&E) for morphological investigations.

Mass Spectrometry

Sample preparation for LC-MS/MS

Liver tissues were resuspended in four volumes of PBS, homogenized with a dounce homogenizer and centrifuged at 1'400 x g for five min. at 4°C. The supernatant was centrifuged at 100'000 x g for one hour at 4°C yielding a soluble and membrane proteome. Proteins were precipitated by the addition of four volumes of methanol, one volume of chloroform, and three volumes of water. Samples were centrifuged at 20'000 x g for five min. at 4°C and the methanol/water and chloroform layers were removed. Proteins were washed with four volumes of methanol and centrifuged once more. Proteins were dried and resuspended in PBS. Proteomes (15 µg) were denatured with 6 M urea in 50 mM NH_4HCO_3, reduced with 10 mM TCEP for 30 min. and alkylated with 25 mM iodoacetamide for 30 min. in the dark. Samples were diluted to 2 M Urea with 50 mM NH_4HCO_3, and digested with trypsin (Promega, 1 µL of 0.5 µg/µL) in the presence of 1 mM $CaCl_2$ for 12 hours at 37 °C. Samples were acidified to a final concentration of 5% acetic acid, desalted over a self-packed C18 spin column, and dried. Samples were analyzed by LC-MS/MS (see below) and the MS data was processed with MaxQuant (see below).

LC-MS/MS analysis

Peptides were resuspended in water with 0.1% formic acid (FA) and analyzed using Proxeon EASY-nLC 1000 nano-UHPLC coupled to QExactive Plus Quadrupole-Orbitrap mass spectrometer (Thermo Scientific). The chromatography column consisted of a 30 cm long, 75 µm i.d. microcapillary capped by a 5 µm tip and packed with ReproSil-Pur 120 C18-AQ 2.4 µm beads (Dr. Maisch GmbH). LC solvents were 0.1% FA in water (Buffer A) and 0.1% FA in acetonitrile (Buffer B). Peptides were eluted into the mass spectrometer at a flow rate of 300 nL/min. over a 240 min. linear gradient (3-35% Buffer B) at 65°C. Data was acquired in data-

dependent mode (top-20, NCE 30, R = 17'500) after full MS scan (R = 70'000, m/z 400-1'300). Dynamic exclusion was set to 10 s, peptide match to prefer and isotope exclusion was enabled.

MaxQuant analysis

The MS data was analysed with MaxQuant(3) (V1.5.2.8) and searched against the mouse proteome (Uniprot) and a common list of contaminants (included in MaxQuant). The first peptide search tolerance was set to 20 ppm, 7 ppm was used for the main peptide search and fragment mass tolerance was set to 0.02 Da. The false discovery rate for peptides, proteins, and sites identification was set to 1%. The minimum peptide length was set to 6 amino acids and peptide re-quantification, label-free quantification (MaxLFQ), and "match between runs" were enabled. The minimal number of peptides per protein was set to two. Oxidized methionines and N-terminal acetylation were searched as variable modifications and carbamidomethylation of cysteines was searched as a fixed modification.

Bioinformatics analyses

Identification of lipid/glucose metabolism-associated proteins differentially expressed in the proteomic analysis and potentially regulated by miR-22-3p

The list of upregulated proteins in miR22KO mice identified in our proteomic analysis was crossreferenced with a list of potential mouse miR-22-3p targets retrieved from the miRWalk 2.0 database. The 54 candidates identified were then segregated by biological processes based on literature. Finally, protein candidates involved in lipid or glucose metabolism were represented in a heatmap (Log2 fold Change) with a hierarchical clustering for the samples based on the Euclydian distance, using Multiple Viewer experiment (MeV) software.

GEO Datasets Analyses

The Gene Expression Omnibus database (http://www.ncbi.nlm.nih.gov/gds/?term=hCC) was used to retrieve transcriptomic datasets of human or mouse models of NAFLD. Fold Change differences and statistical differences between experimental groups were calculated with the GEO2R tool. The results were annotated significant if the p-value was lower to 0.05.

Gene Ontology enrichment analysis

Overrepresented GO terms (biological processes or tissue expression) were identified using DAVID 6.7 database (https://david.ncifcrf.gov/). Functional enrichment analyses were performed on potential miR-22-3p targets or differentially expressed proteins between control and miR22KO mice fed HFD. Enrichments are represented with an EASE score (Modified Fisher's exact test). GO terms having a p-value<0.05 were considered significant.

Expression of miRNA in liver tissues and hepatic cell lines.

The miRmine database (http://guanlab.ccmb.med.umich.edu/mirmine/) was used to retrieve the expression levels of miRNAs in human liver tissues and HepG2 cells. Relative miRNA expressions are expressed as Reads Per Million Reads (RPM).

List of predicted and validated miR-22-3p targets

The miRWalk 2.0 database (http://zmf.umm.uni-heidelberg.de/apps/zmf/mirwalk2/custom.html) was used to retrieve the list of predicted and validated miR-22-3p targets in human and mouse. Only targets recognized by at least 3 different databases were considered for further analyses.

Interactome analysis.

The interactions between proteins deregulated between control and miR-22KO mice fed HFD (proteomic analysis) were determined using the STRING (http://string-db.org) database.

Western Blot Analyses

Tissues were homogenized with tissue lyser II (Qiagen®, Germany) and cells were lysed in ice-cold RIPA buffer. Equal amounts of proteins were resolved by 5-20% gradient SDS-PAGE and blotted to nitrocellulose membranes. Proteins were detected with specific primary antibodies and HRP-conjugated secondary antibodies using enhanced chemoluminescence (ECL select, Amersham, GE healthcare, UK) (List of antibodies in Supplementary Table). Chemoluminescence signal was detected using PXi/PXi Touch from Syngene (Synoptics group, Cambridge, UK). Quantifications were performed using the Syngene Software.

Cell Culture and Transfections

Hepatic cancer cell lines were cultured in DMEM, 10% of foetal bovine serum at 37°C and 5% CO_2. Transfection of 20nM of control and hsa-miR-22-3p mimic nucleotides (miridian, Dharmacon, Colorado, USA) was performed using Viromer® blue (Lipocalyx, Germany).

References

1. Pitetti JL, Calvel P, Romero Y, Conne B, Truong V, Papaioannou MD, et al. Insulin and IGF1 receptors are essential for XX and XY gonadal differentiation and adrenal development in mice. PLoS Genet. 2013;9(1):e1003160.
2. Peyrou M, Bourgoin L, Poher AL, Altirriba J, Maeder C, Caillon A, et al. Hepatic PTEN deficiency improves muscle insulin sensitivity and decreases adiposity in mice. J Hepatol. 2015;62(2):421-9.
3. Cox J, Hein MY, Luber CA, Paron I, Nagaraj N, Mann M. Accurate proteome-wide label-free quantification by delayed normalization and maximal peptide ratio extraction, termed MaxLFQ. Molecular & cellular proteomics : MCP. 2014;13(9):2513-26.

TABLE 1: PRIMERS LIST

Gene	Forward	Reverse
Acadm	TGACGAGTATGTTATCAACGG	CCAACAAGAAATACCAGTTGG
Acaca	GGACACCAGTTTTGCATTGA	AGTTTGGGAGGACATCGAAA
Acox-1	CATGAATCCCGATCTGCG	TCAAGTTCTCGATTTCTCGAC
Cd36	GTCTATCTACGCTGTGTTCG	ACAGGCTTTCCTTCTTTGC
Cpt1-a	ATGGCAGAGGCTCACCAAGC	GATGAACTTCTTCTTCCAGGAGTGC
Fabp1	AAAGTCAAGGCAGTCGTC	CCCAATGTCATGGTATTGGT
Fasn	AAGTTGCCCGAGTCAGAGAACC	ATCCATAGAGCCCAGCCTTCCATC
Fatp1	CGCTTTCTGCGTATCGTCTG	GATGCACGGGATCGTGTCT
Fatp2	GATACTTTCCGGTGGAAAGG	TTCGACCCTCATGACCTG
Fatp3	GCTTTACAAGCACATCTTCC	CTCACCTGGAGATGTGGT
Fbp1	AGCTCTATGGTATCGCTGG	TCTTCAGACACAAGAACACAG
Gck	GCGGAGATGCTCTTTGAC	GTCCCACGATGTTGTTCC
Pkm2	TCGCATGCAGCACCTGATT	CCTCGAATAGCTGCAAGTGGTA
Ppara	ACGATGCTGTCCTCCTTGATG	GTGTGATAAAGCCATTGCCGT
Hk2	TGATCGCCTGCTTATTCACGG	AACCGCCTAGAAATCTCCAGA
Pfkl	CAGCTACGTGAAGGATCTG	CCATACCCATCTTGCTACTC
Pklr	CTTTGCCTCCTTTGTACGA	TCATCAAACTTCTTCACGCC
miR-16	ACAGCCTAGCAGCACGTAAAT	GAGGTATTCGCACCAGAGGA
miR-22	GCCTGAAGCTGCCAGTTGA	GAGGTATTCGCACCAGAGGA
Scd1	TGCTCCAAGAGATCTCCAG	GGAACCAGTATGATCCCG
sno-202	CCTGTGTACTGACTTGATGAAAG	GAGGTATTCGCACCAGAGGA
**univ-RT-polyT**	GAGGTATTCGCACCAGAGGATTTTTT TTTTTTTTTVN	
Shpk (mouse)	CAG GCC AAG GCT GTG AAT	GCC AGC TGC ATC ATA GGA CT
Eno1 (mouse)	AGT ACG GGA AGG ACG CCA CCA	GCG GCC ACA TCC ATG CCG AT
Shpk (human)	GCC AAA GCT GGA ACG TAG AG	CGA GGT GCT GAT GTT GAG AA
Eno1 (human)	GCC TCC TGC TCA AAG TCA AC	AAC GAT GAG ACA CCA TGA CG

TABLE 2: ANTIBODIES LIST

Antibody	Provider	Reference
**ACC**	Cell Signaling Technology, Inc. (Danvers, MA)	3662
**Akt**	Cell Signaling Technology, Inc. (Danvers, MA)	9272
**CD36**	Abcam plc (Cambridge, UK)	ab133625
**ERM**	Cell Signaling Technology, Inc. (Danvers, MA)	3142 S
**FASN**	Cell Signaling Technology, Inc. (Danvers, MA)	3189
**pAKT (ser473)**	Cell Signaling Technology, Inc. (Danvers, MA)	9271
**pAKT (thr308)**	Cell Signaling Technology, Inc. (Danvers, MA)	9275
**SCD1**	Cell Signaling Technology, Inc. (Danvers, MA)	2438
**β-actin**	Cell Signaling Technology, Inc. (Danvers, MA)	4970

TABLE 3: MICE DIETS

Diet	Metabolized energy	Provider	Catalog number
Standard	5% cal/fat	ssniff Spezialdiäten GmbH (Soest, Germany)	E15000-04, EF R/M Control
High-fat	60% cal/fat	ssniff Spezialdiäten GmbH (Soest, Germany)	E15741-34, EF D12492 (II) mod. 60 kJ% Fat, 21kJ% carbohydrates and 19kJ% proteins

The precise molecular content of the diets can be found at: https://www.bio-services.nl/cms/files/Groep2_loboratoriumvoeders/10_catalogue_EF_new_Experimentele_voeders.pdf .

TABLE 4: DEVICES AND SOFTWARES

Experiment	Device	Provider
**Echo-MRI**	EchoMRI-700 quantitative nuclear magnetic resonance analyzer	Echo Medical Systems (Houston, TX)
**Metabolic cages**	LabMaster system	TSE Systems GmbH (Bad Homburg, Germany)
**Plasma analyses**	Abott Architect analyzer	Abott Architect (Paris, France)
**qRT-PCR**	StepOne plus PCR system	Thermo Fisher Scientific (Waltham, MA)
**Western blot analyses**	Genesys/Genetools software	Syngene (Cambridge, UK)
**Western blot acquisition**	PXi gel imaging system	Syngene (Cambridge, UK)

TABLE 5: OTHER REAGENTS

Name	Provider	Catalog number
Complete EDTA-free inhibitors	Roche (Basel, Switzerland)	11873580001
ECL reagent	Amersham (Little Chalfont, Royaume-Uni)	RPN2135
Eosine Y (yellow)	Sigma-Aldrich (St-Louis, MO)	E 4382
FastStart Universal SYBR Green Master (Rox)	Roche (Basel, Switzerland)	O4913914001
FCS	Thermo Fisher Scientific (Waltham, MA)	10270106
Glucotrend Active	Roche (Basel, Switzerland)	4454308
glutamax	Thermo Fisher Scientific (Waltham, MA)	35050.038
Hematoxylin	Merck Millipore (Burlington, MA)	1.0402.0025
Insulin (Actrapid® HM)	Novo Nordisk (Bagsvaerd, Denmark)	44610
Isofluorane	Rothacher & Partner (Berne, Switzerland)	ISO250
NitroCellulose membranes	Amersham (Dübendorf, Switzerland)	RPN303D
Penicillin-Streptomycin	Invitrogen (Carlsbad, CA)	15140122
High capacity RNA to cDNA kit	Life Technologies (Carlsbad, CA)	4387406
Trizol Reagent	Ambion (Carlsbad, CA)	15596-018

DMEM, 1g/L Glucose	Thermo Fisher Scientific (Waltham, MA)	21885025
Mouse insulin Elisa	Mercodia (Sweden)	10-1247-01
Mouse C-peptide Elisa	Merck Millipore (Burlington, MA)	EZRMCP2-21K
TG Roche/Hitachi R1	Roche (Basel, Switzerland)	5171407190
hsa-miR-22 miridian	Dharmacon (Lafayette, CO)	C-300493-03-0002

LEGENDS OF SUPPLEMENTARY FIGURES

Supplementary figure S1. Human miRNA expression in the liver

(A) The miRmine database was used to retrieve the list of miRNAs expressed in human liver tissues. The top 20 miRNAs are represented. miR-22-3p represent the 4th most expressed miRNA. (B) Comparison of the expression levels between miR-22-3p and -5p in human liver tissues. For panel A and B, expression levels are represented as Reads per Million Reads (RPM).

Supplementary figure S2. Gene Ontology enrichment analysis for KEGG pathway

(A, top 20 pathways) or (B) tissue expression with validated and predicted miR-22-3p targets in human and mouse.

Supplementary figure S3. Liver histology of different hepatic steatosis mouse models

H&E staining of liver sections from 2-months-old *db/db, ob/ob* or 4-months old LPTENKO and their corresponding controls. The expression of miR-22-3p in the livers of these mice is presented in figure 1G.

Supplementary figure S4. miR-22-3p expression in Mouse Primary Hepatocytes under lipid exposure

(A) miR-22-3p expression in mouse primary hepatocytes (MPH) isolated from C57BL/6 mice and exposed 48h 50µM of oleic, palmitic or linolenic acid (n=3). Data are means ± SEM. (B) Analysis of miR-22-3p expression in two transcriptomic datasets from the GEO database: SK-Hep1 cells treated with fatty acids and HPH treated with fatty acids and TNFα. *P<0.05, **P<0.01, ***P<0.001 compared with controls (Student t-test for panel A and one-way ANOVA for panels B and C).

Supplementary figure S5. Strategy for development of miR-22KO mice

(A) Illustration of the *Mir22* gene locus (wt allele), the targeted locus with loxP sites inserted (*Mir22* floxed allele), and the locus with *Mir22* deleted (miR-22 KO allele). (B) Quantitative RT-PCR analysis of mature miR-22-3p expression in the liver, epididymal white adipose tissue and gastrocnemius of CTL and miR-22KO mice. (n=3-10, data are means±SEM)

Supplementary figure S6. Autopsy representation of WT and miR22KO mice under HFD

Representative pictures of autopsies from control or miR-22KO mice fed a HFD for 12 weeks.

Supplementary figure S7. Energy expenditure in CTL and miR-22KO mice fed a high fat diet. (A) Respiratory Exchange Ratio (RER), (B) Fuel oxidation with the respective contributions of fat and carbohydrate/protein oxidation, (C) Food consumption, (D) water consumption, and (E) Locomotor activity. (n=6, data are represented as means ± SEM).

Supplementary figure S8. Lipid synthesis and trafficking-related proteins in the eWAT
Representative Western blots (n=5 for control mice and n=for miR22KO mice) and quantifications (N=5-6 mice per group, data are means ± SEM) of protein expressed in epididymal white adipose tissue and involved in *de novo* lipogenesis (ACC, FASN and SCD1) and fatty acid traficking (CD36) in CTL and miR-22KO mice fed a high fat diet. The protein contents of CD36, ACC, FASN and SCD1 were reported to the β-actin content in each mouse. *P<0.05, **P<0.01, ***P<0.001 compared with controls (Student t-test).

Supplementary figure S9. Insulin signaling-related proteins in the eWAT, mice fed HFD
Western Blot analyses of phospho-AKT (serine and threonine residues), AKT, insulin receptor (IR) and phospho-IR in eWAT from starved mice injected with either NaCl or insulin (150mU/g) for 40 minutes (n=2-3 different mice for control groups treated or not with insulin, and n=3 different mice for miR-22KOmice treated or not with insulin, data are means±SEM). The content of phospho-IR was reported to the total IR content and the phosphoThr-AKT and phosphoSer-AKT were reported to the total AKT content in each mouse. *P<0.05, **P<0.01, ***P<0.001 compared with controls (Student t-test)

Supplementary figure S10. Liver histology of miR-22KO mice under CD
Representative Hematoxilin/eosin staining of liver sections from control or miR-22KO mice fed a chow diet for 12 weeks.

Supplementary figure S11. Lipid catabolism enzymes in the liver of miR-22KO mice
RT-qPCR analysis of lipid catabolism related enzymes in the liver of miR-22KO mice and their respective controls after 12 weeks of HFD. (n=6 mice per group, data are means ± SEM).

Supplementary figure S12. miR-22-3p expression in various cell lines
(A) Expression level of miR-22-3p in human primary hepatocytes (HPH) and human hepatic cancer cell lines (HepaRG, Huh-7 and HepG2). Data are represented as Fold Change relative to HPH. (B) The miRmine database was used to retrieve the expression level of miRNAs in HepG2 cells. Expression levels are represented as Reads per Million Reads (RPM). (C) RT-qPCR analysis of glycolytic gene expression in Huh7 cells transfected with miR-22-3p

oligonucleotides (20nM, 48h transfection, n=6). Data represent the mean ±SEM. *P<0.05, **P<0.01, ***P<0.001 compared with controls (One-way ANOVA).

Supplementary figure S13. Validated lncRNA targeting miR-22-3p (miRwalk/Literature) (A) A list of validated long-non-coding RNA (lncRNA) targeting miR-22-3p was made by using miRWalk 2.0 database and a literature screening. lncRNA predicted by miRWalk 2.0 are indicated with a *. The corresponding PMIDs and models for each lncRNA are also indicated. (B) Comparison of Predicted and validated lncRNA binding miR-22-3p, based on miRWalk 2.0 database and literature. The total number of validated lncRNAs that interact with miR-22-3p is 12 (blue circle and S13A). Among all experimentally validated lncRNAs interacting with miR-22-3p, only nine of them (intersection of blue and green circle) were predicted by currently publicly available bioinformatic tools. (C) Expression of potential lncRNA involved in miR-22-3p regulation in various human/mouse models of NAFLD or HCC. Corresponding PMIDs for each lncRNA are indicated.

A

Human miRNA expression in the liver (miRMine database: top 20 miRs)

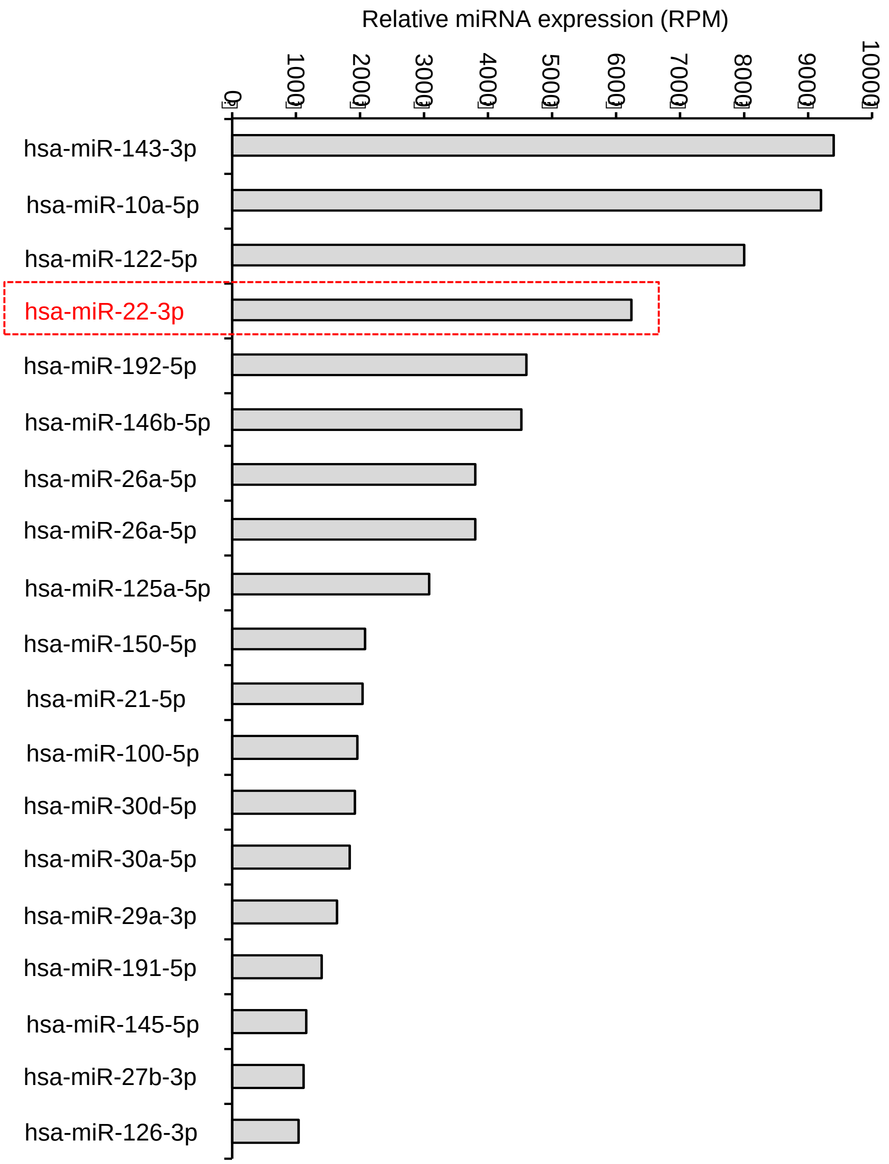

B

miRMine database: mir-22-3p and mir-22-5p in Human liver

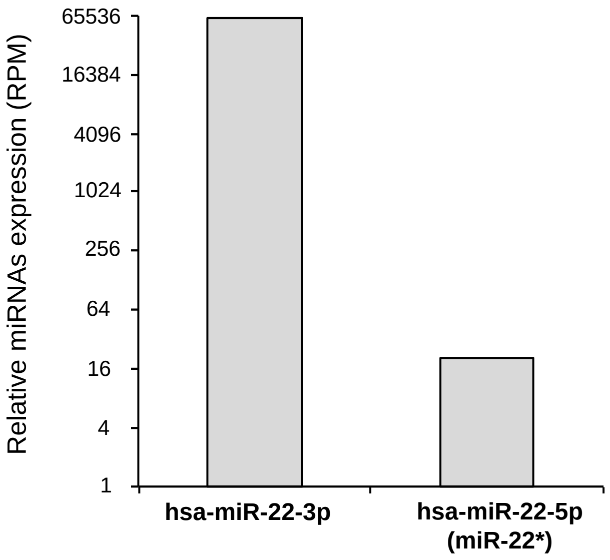

S1

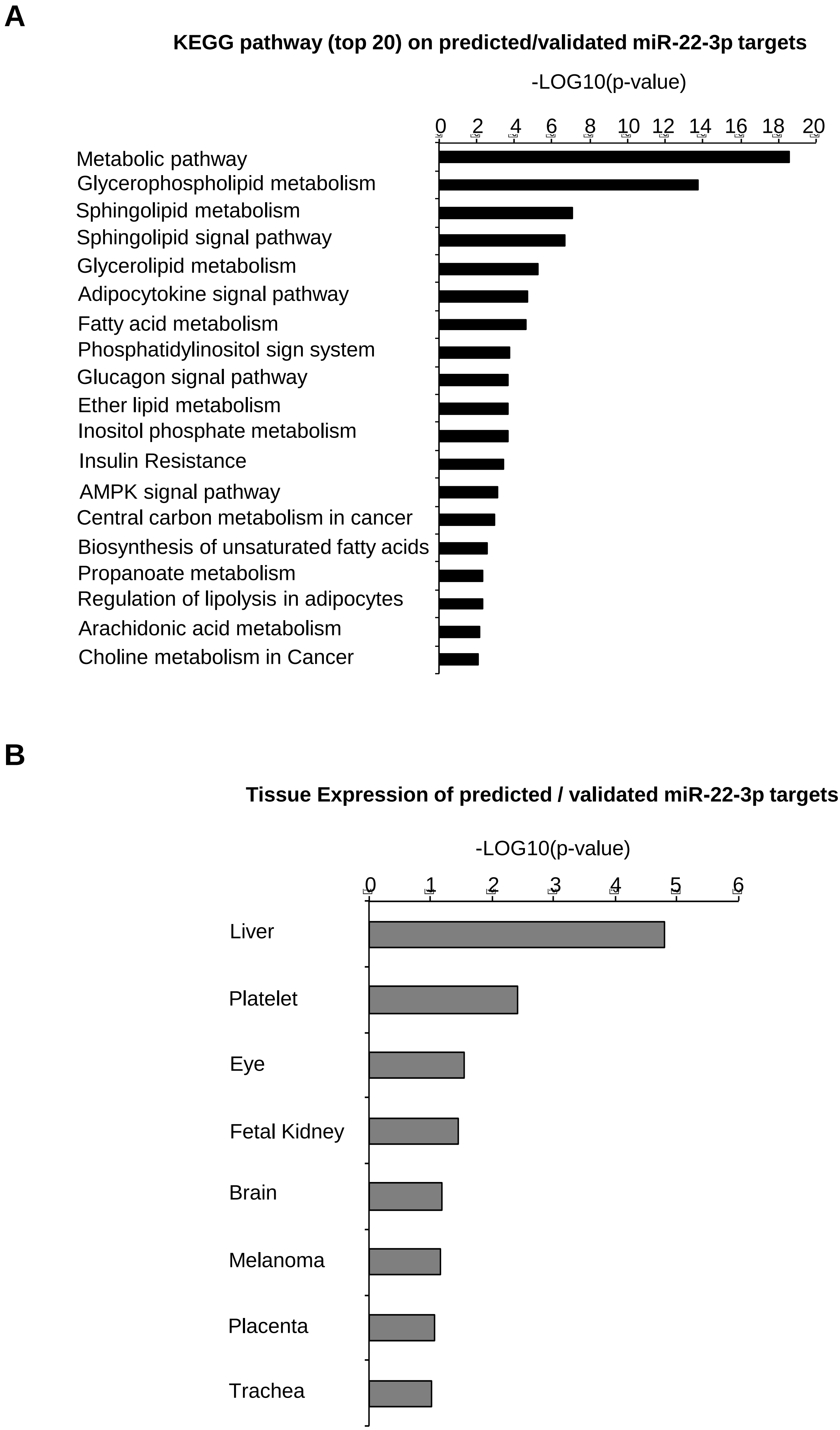
A
KEGG pathway (top 20) on predicted/validated miR-22-3p targets
-LOG10(p-value)
0 2 4 6 8 10 12 14 16 18 20
Metabolic pathway
Glycerophospholipid metabolism
Sphingolipid metabolism
Sphingolipid signal pathway
Glycerolipid metabolism
Adipocytokine signal pathway
Fatty acid metabolism
Phosphatidylinositol sign system
Glucagon signal pathway
Ether lipid metabolism
Inositol phosphate metabolism
Insulin Resistance
AMPK signal pathway
Central carbon metabolism in cancer
Biosynthesis of unsaturated fatty acids
Propanoate metabolism
Regulation of lipolysis in adipocytes
Arachidonic acid metabolism
Choline metabolism in Cancer
B
Tissue Expression of predicted / validated miR-22-3p targets
-LOG10(p-value)
0 1 2 3 4 5 6
Liver
Platelet
Eye
Fetal Kidney
Brain
Melanoma
Placenta
Trachea

S2

A

Liver histology of different hepatic steatosis mouse models

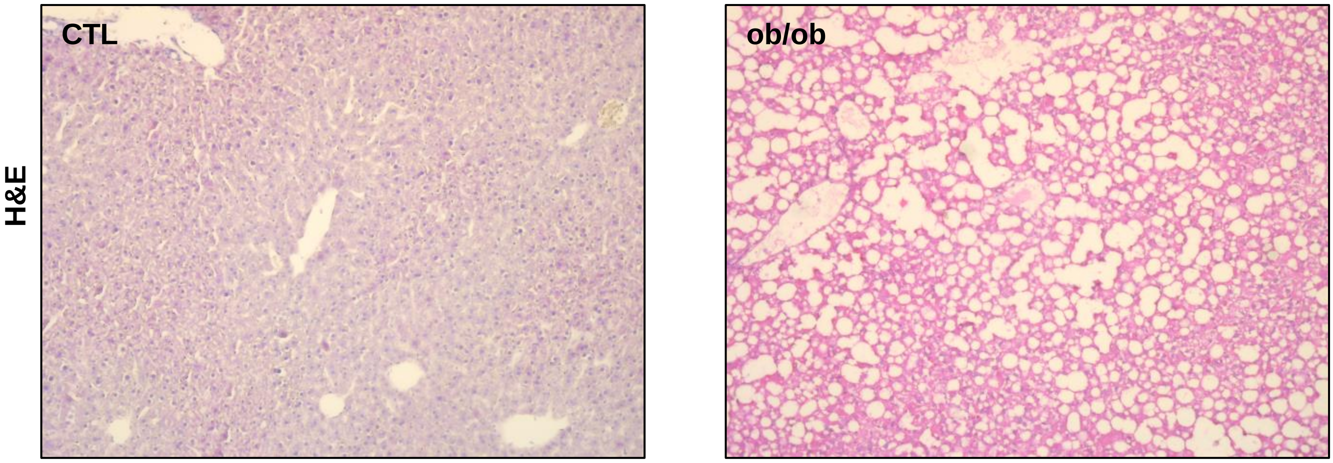

B

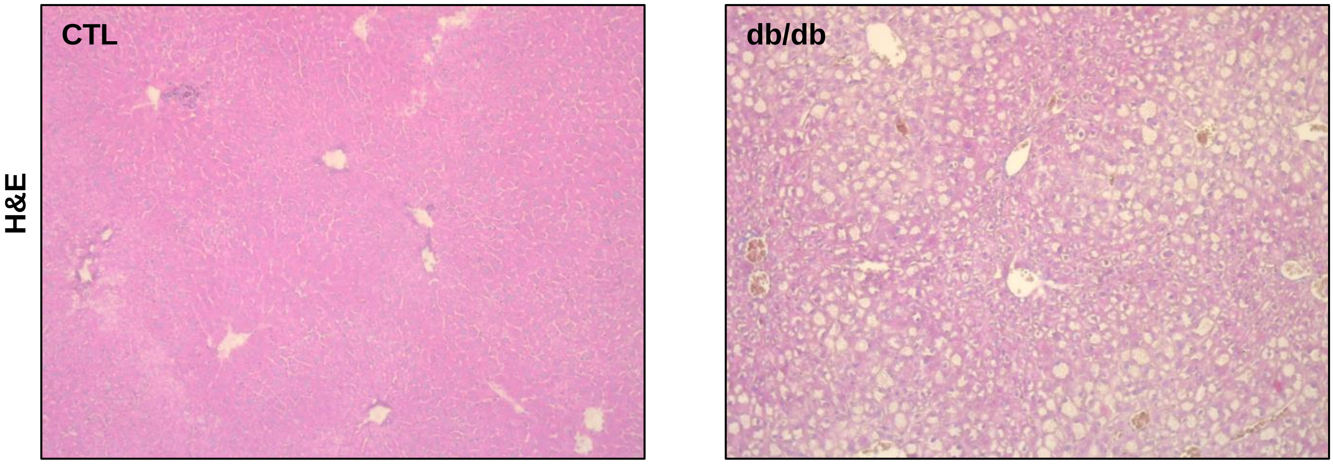

C

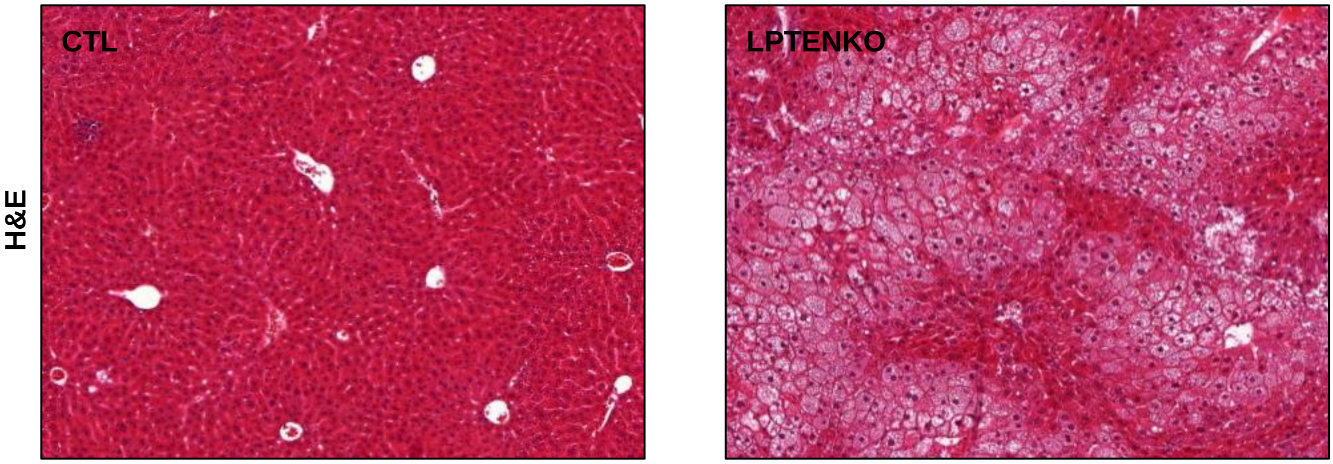

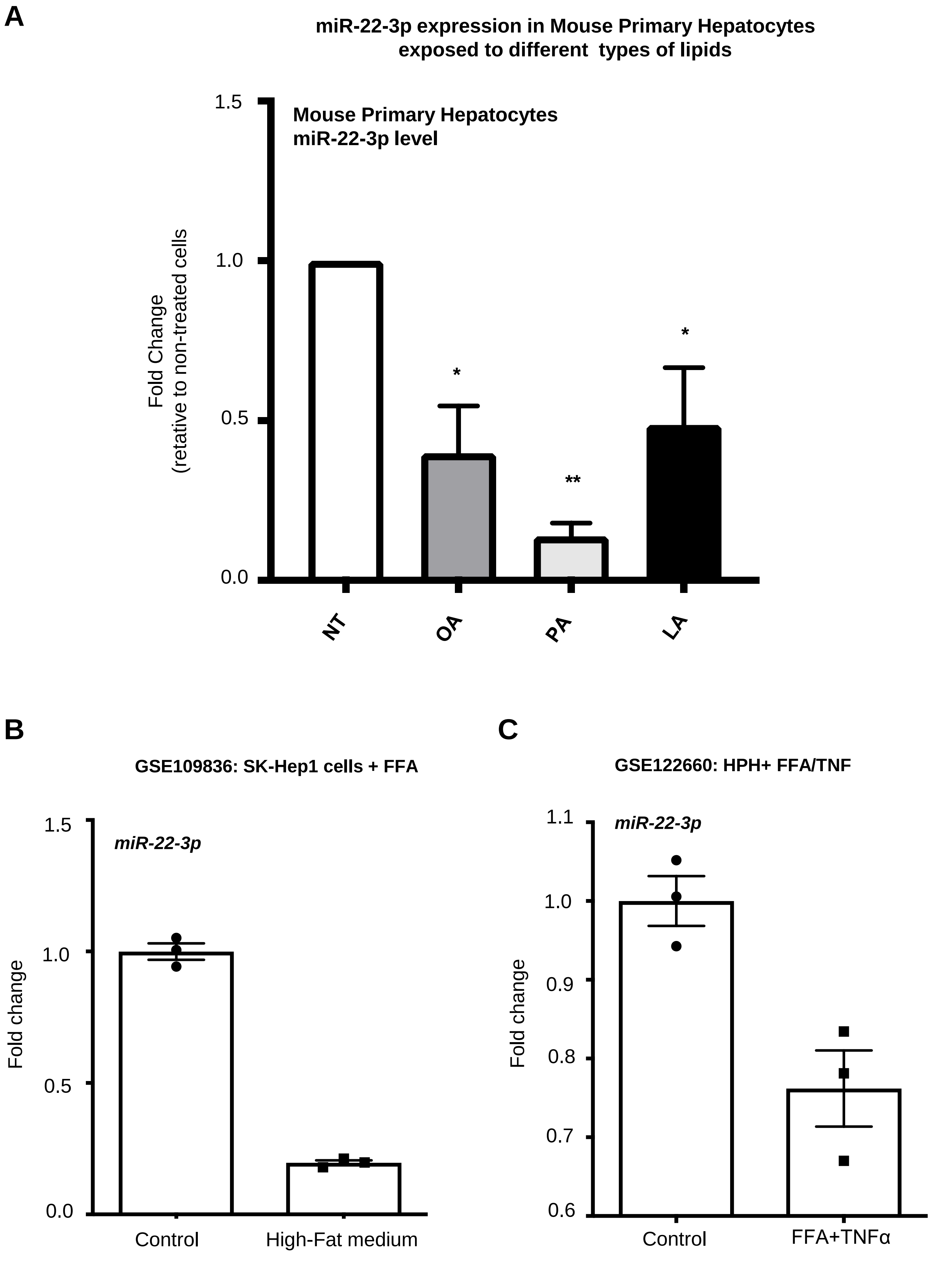
A
miR-22-3p expression in Mouse Primary Hepatocytes
exposed to different types of lipids
Mouse Primary Hepatocytes
miR-22-3p level
Fold Change
(retative to non-treated cells
1.5
1.0
0.5
0.0
*
**
*
NT
OA
PA
LA
B
GSE109836: SK-Hep1 cells + FFA
miR-22-3p
Fold change
1.5
1.0
0.5
0.0
Control
High-Fat medium
C
GSE122660: HPH+ FFA/TNF
miR-22-3p
Fold change
1.1
1.0
0.9
0.8
0.7
0.6
Control
FFA+TNFα

A

Strategy for development of miR-22KO mice

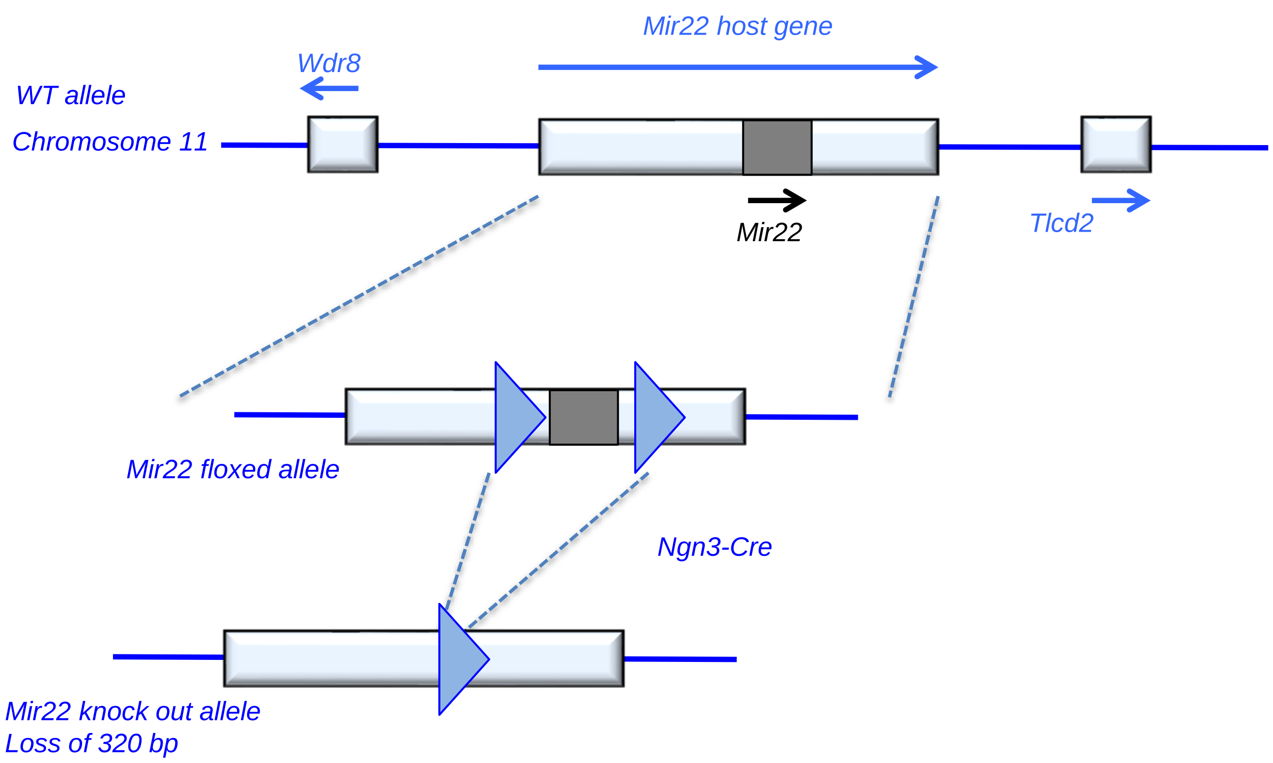

B

Expression of miR-22-3p in different metabolic organs in control and miR-22KO mice

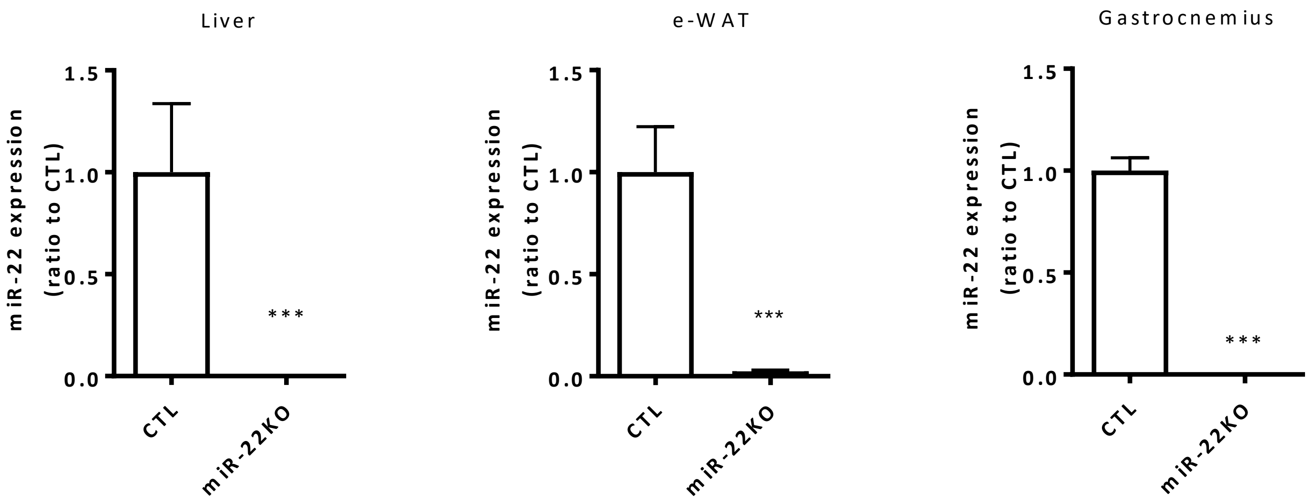

Autopsy representation of WT and miR22KO mice under HFD

CTL

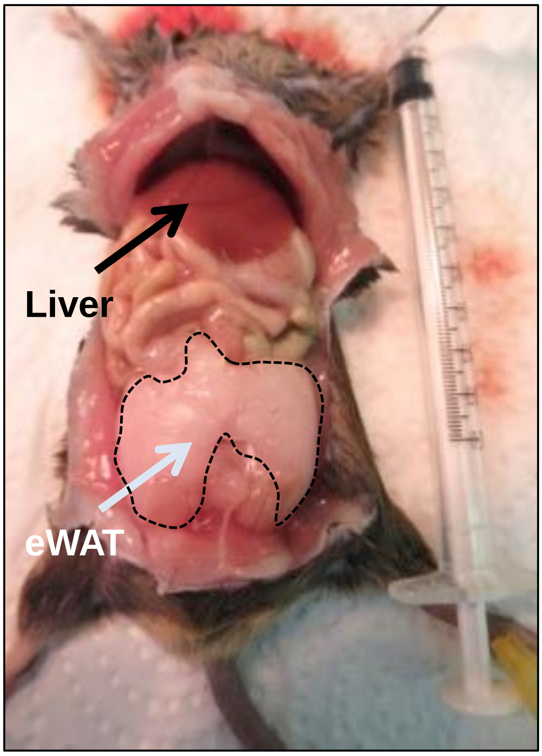

miR-22KO

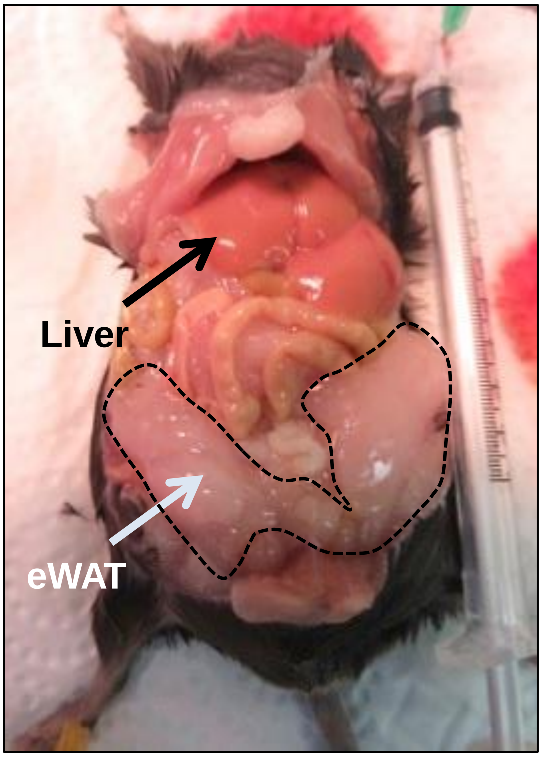

Metabolic parameters of miR-22KO mice

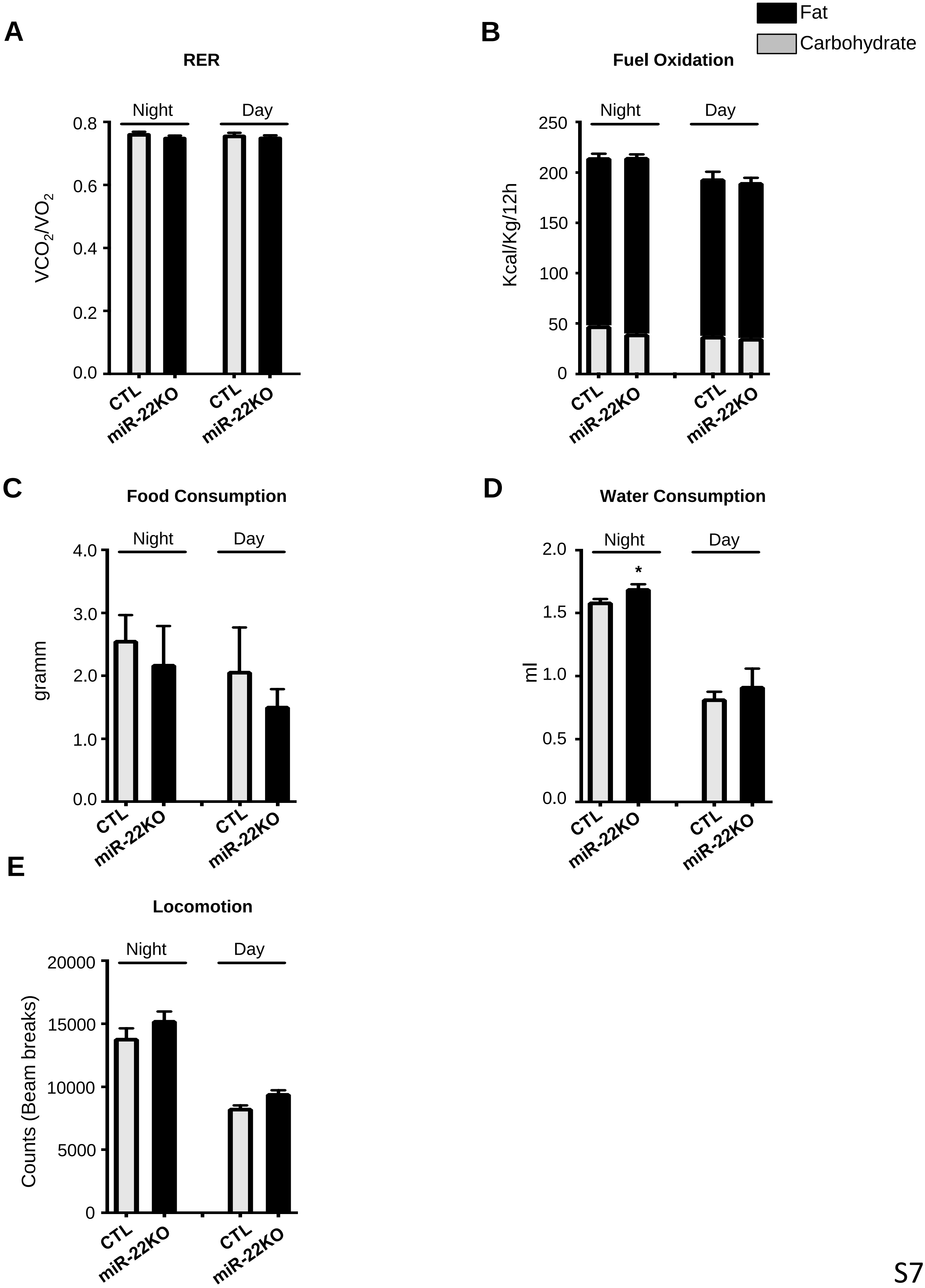

S7

Western blot analysis of lipid synthesis and trafficking-related proteins in the eWAT

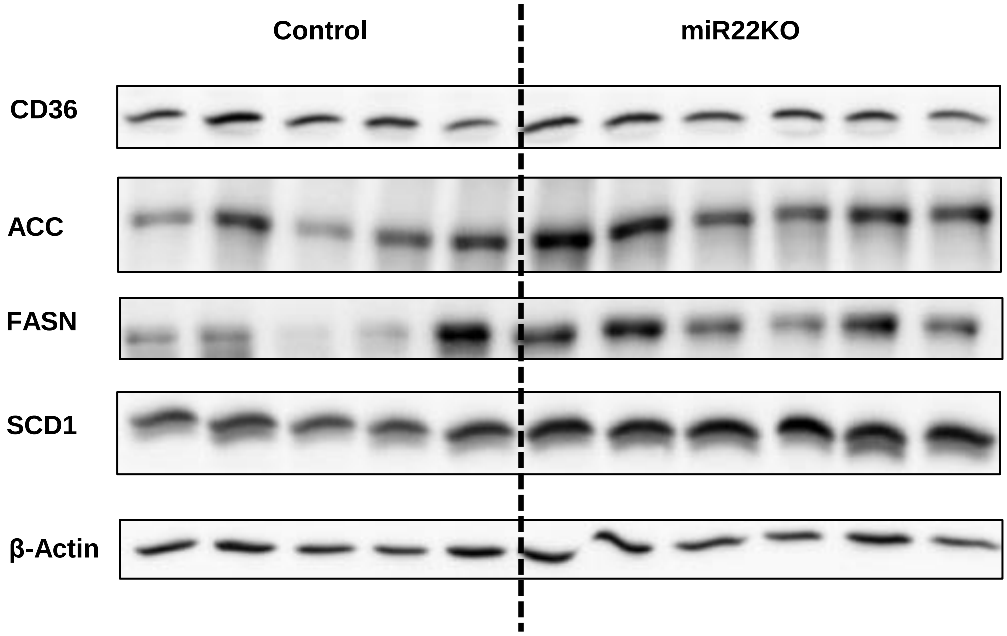

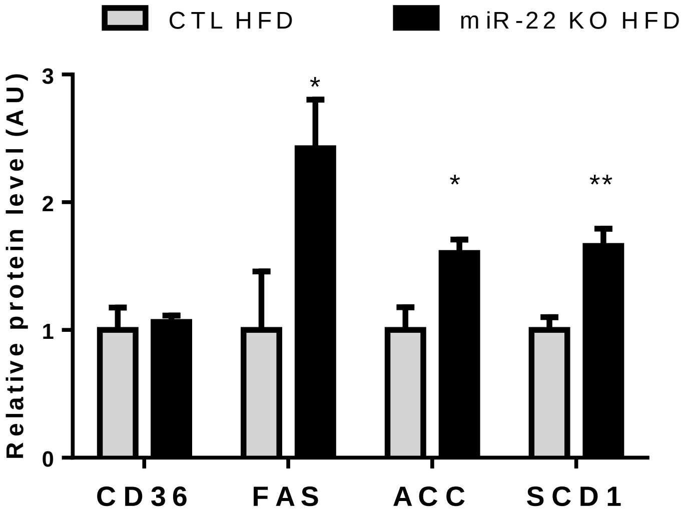

Western blot analysis of insulin signaling-related proteins in the eWAT, mice fed HFD

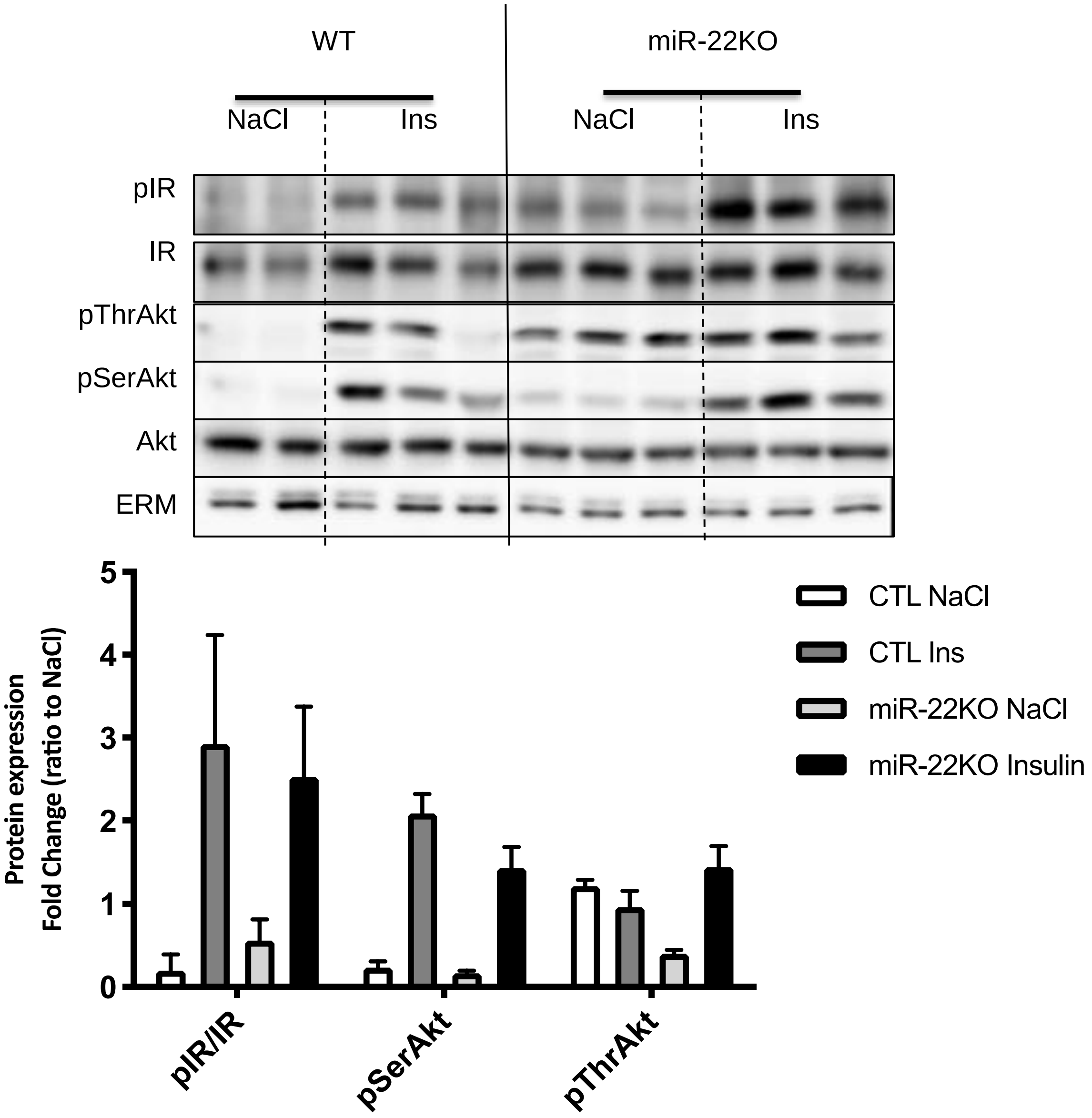

Liver histology of miR-22KO mice under CD

Control liver Chow Diet

H&E staining

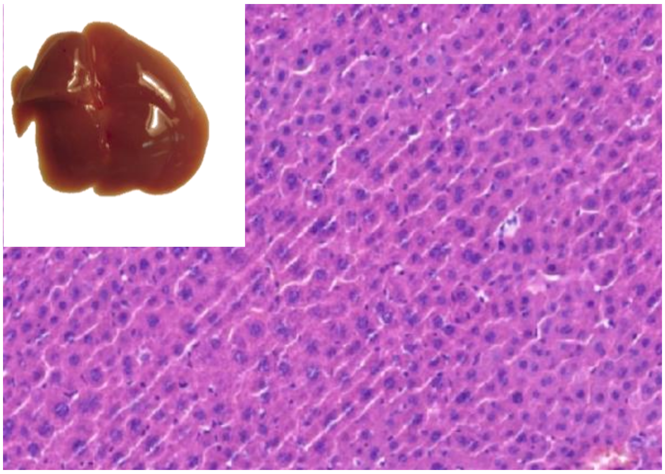

miR-22KO liver Chow Diet

H&E staining

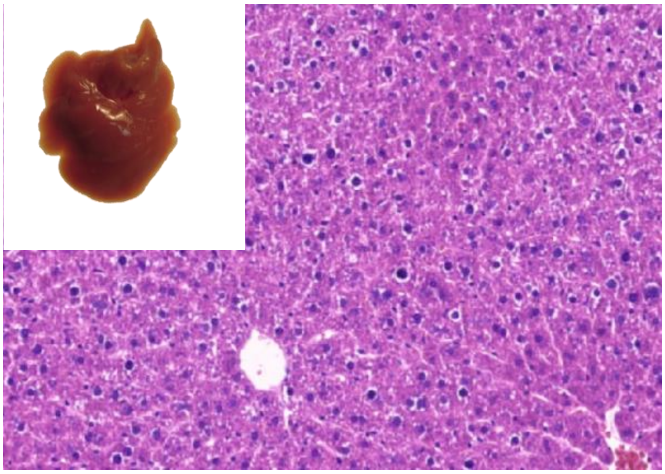

RT-qPCR analysis of lipid catabolism related enzymes in the liver of miR-22KO mice and their respective controls after 12 weeks of HFD

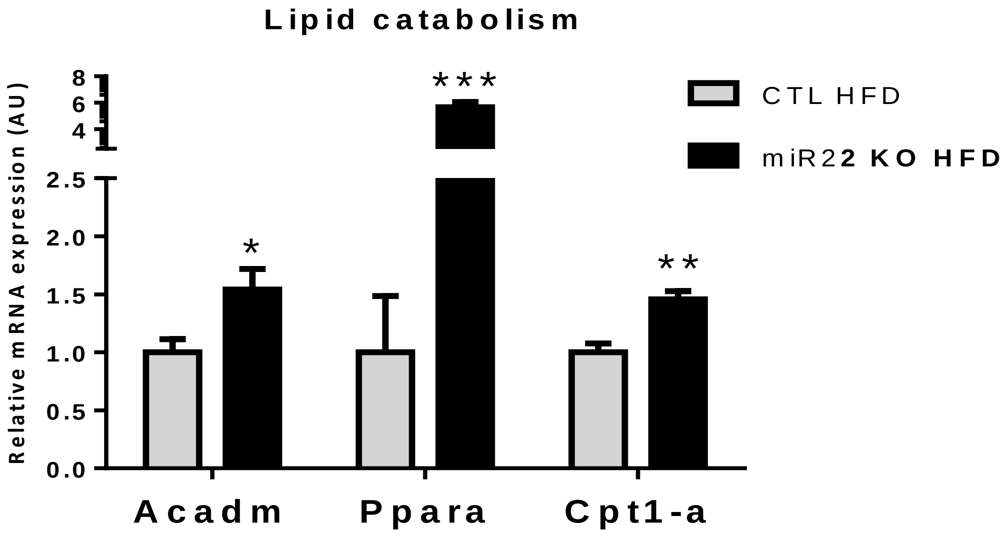

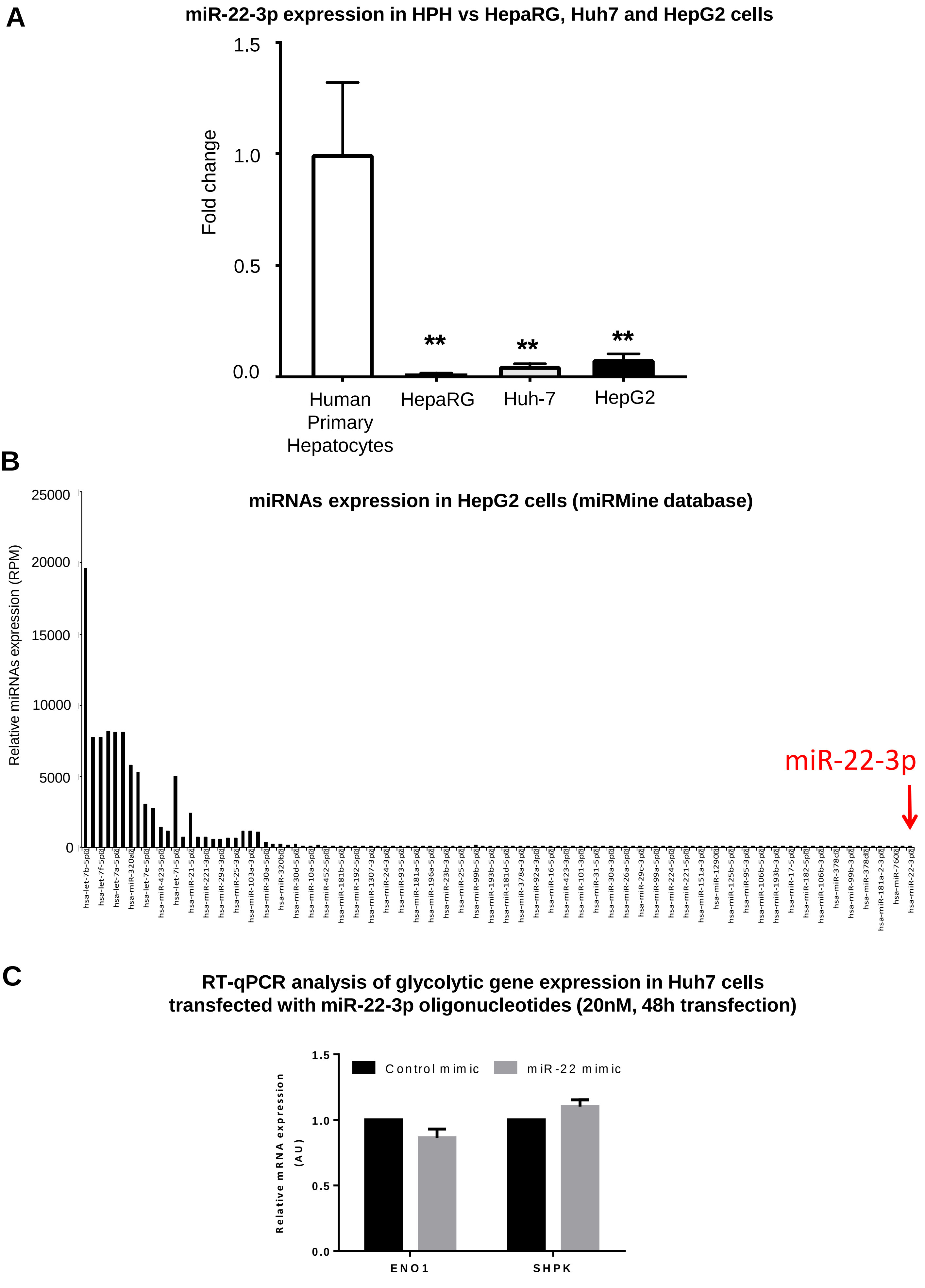
A
miR-22-3p expression in HPH vs HepaRG, Huh7 and HepG2 cells
Fold change
1.5
1.0
0.5
0.0
**
**
**
Human Primary Hepatocytes
HepaRG
Huh-7
HepG2
B
miRNAs expression in HepG2 cells (miRMine database)
Relative miRNAs expression (RPM)
25000
20000
15000
10000
5000
0
miR-22-3p
hsa-let-7b-5p
hsa-let-7f-5p
hsa-let-7a-5p
hsa-miR-320a
hsa-let-7e-5p
hsa-miR-423-5p
hsa-let-7i-5p
hsa-miR-21-5p
hsa-miR-221-3p
hsa-miR-29a-3p
hsa-miR-25-3p
hsa-miR-103a-3p
hsa-miR-30a-5p
hsa-miR-320b
hsa-miR-30d-5p
hsa-miR-10a-5p
hsa-miR-452-5p
hsa-miR-181b-5p
hsa-miR-192-5p
hsa-miR-1307-3p
hsa-miR-24-3p
hsa-miR-93-5p
hsa-miR-181a-5p
hsa-miR-196a-5p
hsa-miR-23b-3p
hsa-miR-25-5p
hsa-miR-99b-5p
hsa-miR-193b-5p
hsa-miR-181d-5p
hsa-miR-378a-3p
hsa-miR-92a-3p
hsa-miR-16-5p
hsa-miR-423-3p
hsa-miR-101-3p
hsa-miR-31-5p
hsa-miR-30a-3p
hsa-miR-26a-5p
hsa-miR-29c-3p
hsa-miR-99a-5p
hsa-miR-224-5p
hsa-miR-221-5p
hsa-miR-151a-3p
hsa-miR-1290
hsa-miR-125b-5p
hsa-miR-95-3p
hsa-miR-106b-5p
hsa-miR-193b-3p
hsa-miR-17-5p
hsa-miR-182-5p
hsa-miR-106b-3p
hsa-miR-378c
hsa-miR-99b-3p
hsa-miR-378d
hsa-miR-181a-2-3p
hsa-miR-760
hsa-miR-22-3p
C
RT-qPCR analysis of glycolytic gene expression in Huh7 cells
transfected with miR-22-3p oligonucleotides (20nM, 48h transfection)
Relative mRNA expression (AU)
1.5
1.0
0.5
0.0
Control mimic
miR-22 mimic
ENO1
SHPK

A

Validated lncRNA targeting miR-22-3p (miRwalk/Literature)

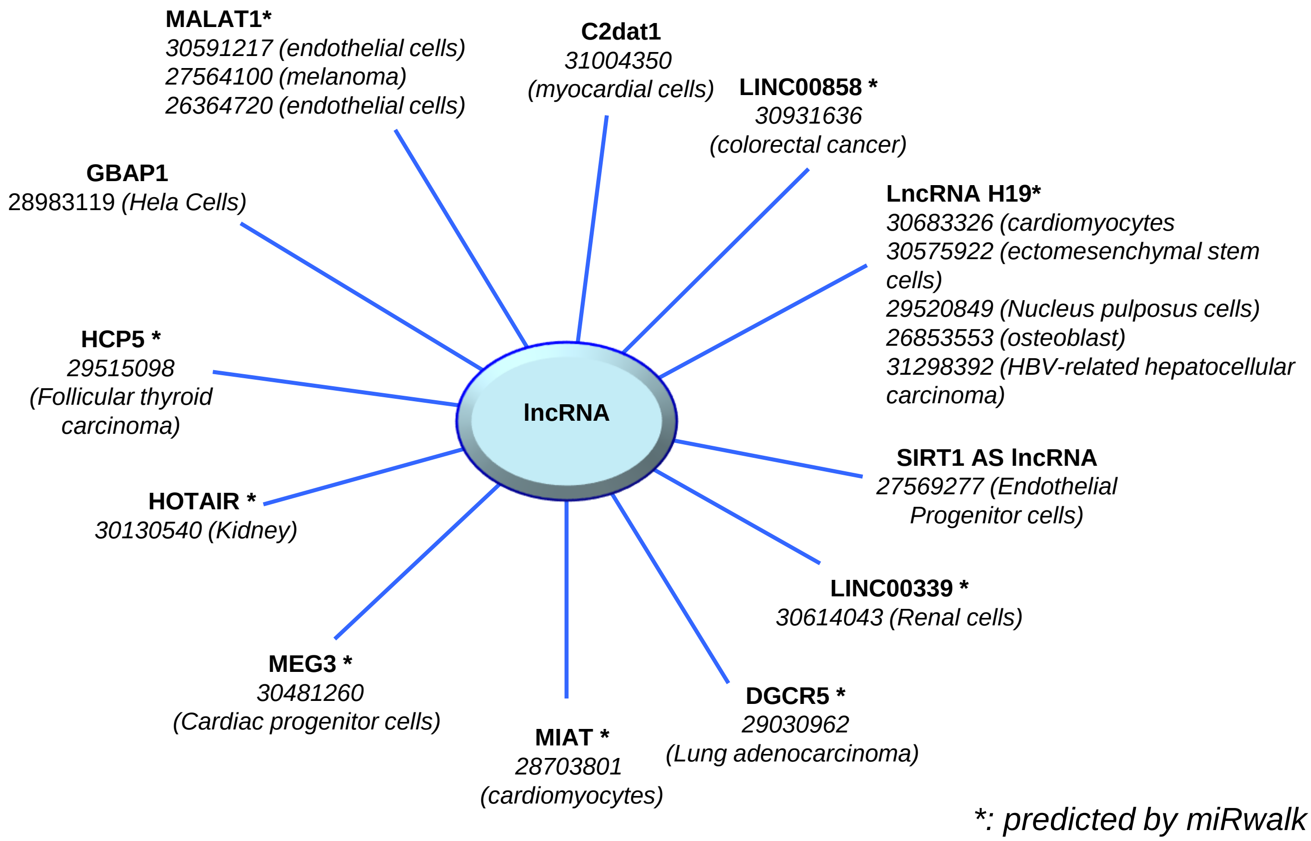

B

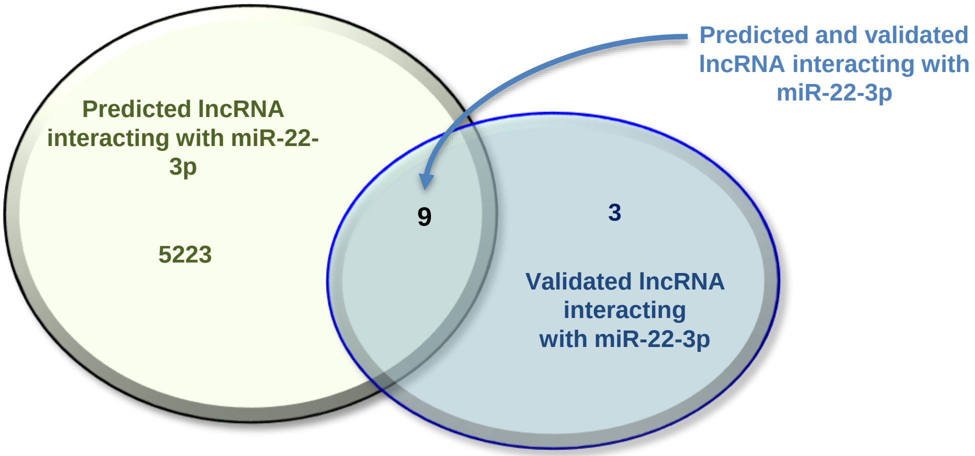

C

lncRNA	Model	Expression	PMIDs
MALAT1	Liver fibrosis	↑	*28993096*
MALAT1	HepG2 +palmitate<br>Liver ob/ob	↑	*26935028*
LncRNA-H19	Mice fed a HFD<br>HepG2/Huh7 cells + FFA	↑	*31064820*
MEG3	Mice fed a HFD<br>HepG2 cells + FFA	↓	*30711569*
LINC00339	HCC tissues and cell lines (Huh7, HepG2, Huh-6, SK-Hep-1)	↑	31081143
